# Content Validity of Patient-Reported Outcome Measures Developed for Assessing Health-Related Quality of Life in People with Type 2 Diabetes Mellitus: a Systematic Review

**DOI:** 10.1007/s11892-022-01482-z

**Published:** 2022-07-11

**Authors:** Caroline B. Terwee, Petra J. M. Elders, Marlous Langendoen-Gort, Ellen B. M. Elsman, Cecilia A. C. Prinsen, Amber A. van der Heijden, Maartje de Wit, Joline W. J. Beulens, Lidwine B. Mokkink, Femke Rutters

**Affiliations:** 1 Amsterdam UMC, Department of Epidemiology and Data Science, Vrije Universiteit Amsterdam, Amsterdam Public Health Research Institute, Amsterdam, Netherlands; 2 Amsterdam UMC, Department of General Practice, Vrije Universiteit Amsterdam, Amsterdam Public Health Research Institute, Amsterdam, Netherlands; 3 Amsterdam UMC, Department of Medical Psychology, Vrije Universiteit Amsterdam, Amsterdam Public Health Research Institute, Amsterdam, Netherlands

**Keywords:** Patient-reported outcomes, Questionnaire, Validity, Diabetes, Systematic review, Quality of life

## Abstract

**Purpose of review:**

We aimed to systematically evaluate the content validity of patient-reported outcome measures (PROMs) specifically developed to measure (aspects of) health-related quality of life (HRQOL) in people with type 2 diabetes. A systematic review was performed in PubMed and Embase of PROMs measuring perceived symptoms, physical function, mental function, social function/participation, and general health perceptions, and that were validated to at least some extent. Content validity (relevance, comprehensiveness, and comprehensibility) was evaluated using COSMIN methodology.

**Recent findings:**

We identified 54 (different versions of) PROMs, containing 150 subscales. We found evidence for sufficient content validity for only 41/150 (27%) (subscales of) PROMs. The quality of evidence was generally very low. We found 66 out of 150 (44%) (subscales of) PROMs with evidence for either insufficient relevance, insufficient comprehensiveness, or insufficient comprehensibility. For measuring diabetes-specific symptoms, physical function, mental function, social function/participation, and general health perceptions, we identified one to 11 (subscales of) PROMs with sufficient content validity, although quality of the evidence was generally low. For measuring depressive symptoms, no PROM with sufficient content validity was identified.

**Summary:**

For each aspect of HRQL, we found at least one PROM with sufficient content validity, except for depressive symptoms. The quality of the evidence was mostly very low.

**Supplementary Information:**

The online version contains supplementary material available at 10.1007/s11892-022-01482-z.

## Introduction

In recent years, the use of patient-reported outcome measures (PROMs) in routine diabetes care has significantly increased. PROMs are questionnaires completed by patients that measure perceived symptoms and the impact of symptoms on physical function, mental function, social function, and general health perceptions (often referred to as (aspects of) health-related quality of life (HRQOL)). PROMs have the potential to harness the voice of patients. They provide clinically important and complementary predictive information regarding effects of interventions, risk of hospitalization, and medication needs, can help clinicians with treatment decision support and monitoring, and help prioritize the use of healthcare resources for optimal public health benefit [1].

Many different PROMs are used in care and research in people with type 2 diabetes, yet no consensus exists regarding which PROMs to use in research or clinical practice. In our recent systematic review, we identified 108 unique PROMs for measuring HRQOL in people with type 2 diabetes, addressing a variety of constructs [2]. The harmonization of PROMs for use in diabetes care and research has been challenged by a lack of conceptual clarity and consensus regarding the core domains and constructs to be measured such as “diabetes-related quality of life” [1]. This heterogeneity hampers the usefulness of PROMs to inform value-based health care and is a serious threat to comparative effectiveness research, despite recent initiatives such as from the International Consortium for Health Outcomes Measurements (ICHOM) and the American Diabetes Association (ADA) to standardized PRO measurements [3, 4].

A good-quality PROM is developed in collaboration with patients to ensure that it measures what is most important to patients. Furthermore, the PROM should have good measurement properties, which means it is valid (it measures what aims to measure), reliable (it gives the same scores on repeated measurements in stable patients), and responsive (it is able to measure change in the PRO over time) (Appendix 1) [5].

A key part of validity is content validity, which is considered the most important measurement property, referring to the relevance, comprehensiveness, and comprehensibility of a PROM (Table 1) [5–8]. Relevance means that all questions (also called items) of a PROM measure things that are relevant for the outcome (also called construct), which the PROM aims to measure. It also means that the PROM does not measure things that are not related to the outcome of interest. For example, if a PROM aims to measure “physical function”, the questions should ask about the capability to perform, or perceived limitations in, relevant activities. The PROM should not include questions about other constructs, such as pain or fatigue. Comprehensiveness means that the PROM should measure all important aspects of the construct of interest; no key aspects should be missing. Furthermore, comprehensibility means that the questions are understood by people who complete them as intended. To be able to test whether a PROM has good content validity, the PROM should have a clear definition of the construct that it aims to measure. If a PROM does not have good content validity, wrong conclusions may be drawn when using that PROM [6].

**Table 1:** Criteria for good content validity [6]

Relevance	
1	Are the included items relevant for the construct of interest?
2	Are the included items relevant for the target population of interest?
3	Are the included items relevant for the context of use of interest?
4	Are the response options appropriate?
5	Is the recall period appropriate?
Comprehensiveness	
6	Are all key concepts included?
Comprehensibility	
7	Are the PROM instructions understood by the population of interest as intended?
8	Are the PROM items and response options understood by the population of interest as intended?
9	Are the PROM items appropriately worded?
10	Do the response options match the question?

High-quality systematic reviews are needed that evaluate and compare the measurement properties of PROMs to select the best PROMs for research or care. At least 16 systematic reviews of PROMs have been published in the field of diabetes [9–24]. However, only seven reviews evaluated content validity of the included PROMs to some extent [10, 12, 13, 18–20, 22]. Five of these reviews did not provide a comprehensive overview of content validity but only evaluated whether people with diabetes were involved in the PROM development [10, 13, 18–20]. One review did not take the quality of the PROM development into account, and results for relevance, comprehensiveness, and comprehensibility were not presented separately, which limits its usefulness for identifying gaps and further development of the PROMs [22]. One review evaluated relevance, comprehensiveness, and comprehensibility separately, but this was only done for PROMs relevant to differentiate effects of oral hypoglycaemic agents [12].

The aim of the present study was to systematically evaluate the content validity of PROMs, which have specifically been developed to measure (aspects of) HRQOL in people with type 2 diabetes. We included PROMs that measured perceived symptoms, physical function, mental function, social function/participation, and general health perceptions and which were validated to at least some extent. We aim to provide evidence-based recommendations for the most suitable PROMs for use as outcome measures in research and clinical practice.

## Methods

### Design

We performed a systematic review using the Consensus-based Standards for the Selection of Health Measurement Instruments (COSMIN) methodology for systematic reviews of PROMs [25] and for assessing content validity [6]. This review was part of a larger project that aimed to identify all PROMs measuring (aspects of) HRQOL used in the field of type 2 diabetes [2]. The protocol was registered in the PROSPERO database: CRD42017071012.

### Literature Search

**Fig. 1:**
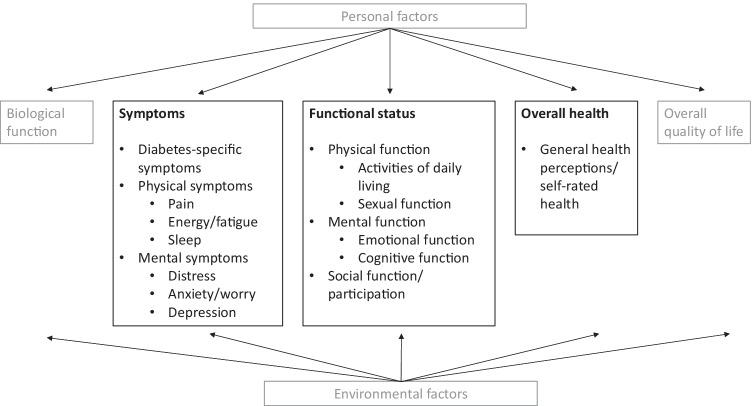
Model of health outcomes based on Wilson and Cleary [72]

The full literature search and data extraction process are described elsewhere [2]. The exact search strategy can be found in Appendix 2. In brief, we searched the databases PubMed and Embase from inception till April 29, 2019. Inclusion criteria for this content validity review were, first, the PROM measures perceived symptoms, physical function, mental function, social function/participation, or general health perceptions (Fig. 1). Second, the PROM was developed specifically for people with type 2 diabetes or for all people with diabetes if at least 50% of the study population consisted of people with type 2 diabetes. Third, the PROM is useful for evaluative purposes (e.g. monitor change over time). Fourth, the aim of the study was the development of a PROM or an evaluation of content validity. Fifth, we also included studies reporting on a pilot study after translation of a PROM because such studies provide evidence for comprehensibility of the PROM. Sixth, we only included full-text papers, in English or Dutch, because detailed understanding of methods used in papers was required and the authors are not proficient in other languages. We excluded PROMs measuring overall quality of life (QOL) and PROMs that were primarily developed for diagnostic, screening, or prognostic purposes.

Each abstract or full-text paper was independently reviewed by two reviewers from the review team. If reviewers disagreed, they discussed the abstract or paper until consensus was reached or a third author with experience in systematic reviews of PROMs made the final decision. References of the included articles were checked by one reviewer to search for additional potentially relevant studies. If information on PROM development was lacking in a paper, we searched Google (manuals or websites) and the PROQOLID database for additional resources.

### Data Extraction

Data extraction on PROM characteristics was performed in the larger review [2]. For this content validity review, characteristics of the study populations included in the PROM development and content validity studies, i.e. age, sex, disease characteristics, setting, country, and language version of the PROM, were extracted by one reviewer.

### Evaluation of Content Validity

We assessed the content validity of the PROMs in three steps, described in detail in Table 2. In step 1, we evaluated the quality of the development study of the PROM, using box 1 of the COSMIN Risk of Bias checklist for PROMs [26]. In step 2, we evaluated the quality of available content validity studies, which were performed after the PROM was developed (external validity), using box 2 of the COSMIN Risk of Bias checklist for PROMs. In step 3, we evaluated the relevance, comprehensiveness, and comprehensibility of the PROMs itself, using the criteria described in Table 1, based on the methods and results of the available PROM development, additional content validity studies if available, and our own rating of the content of the PROM. This was done first per study (step 3a), and subsequently, all available evidence on the relevance, comprehensiveness, and comprehensibility of a specific PROM was summarized and rated as sufficient ( +), insufficient ( −), inconsistent ( ±), or indeterminate (?) (step 3b). Finally, each rating of the content validity per PROM was accompanied by a grade for the quality of the evidence (high, moderate, low, very low), using a modified GRADE approach [27], indicating how confident we are that the ratings are trustworthy (for example, the quality of the evidence was rated higher if the studies were of high-quality or if there was evidence from multiple studies) (step 3c).

**Table 2:**
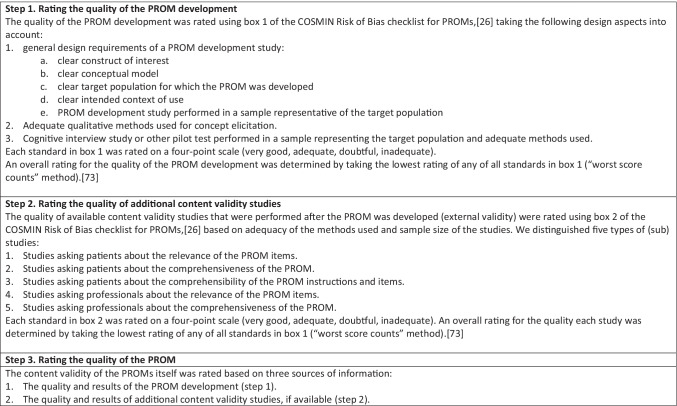

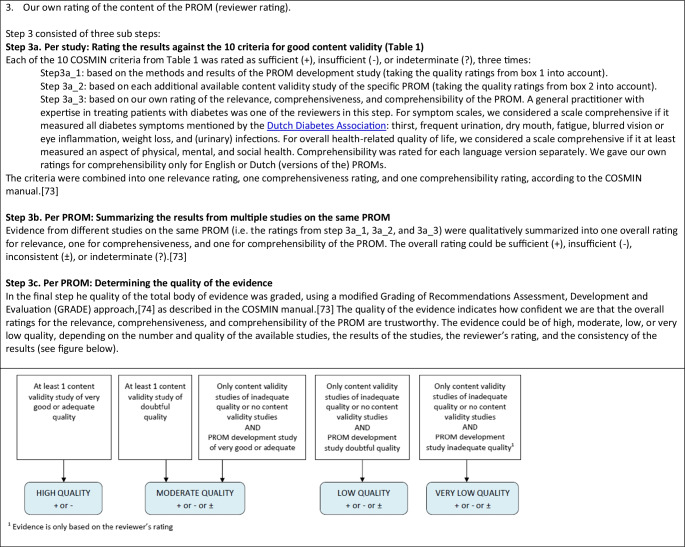
Methodology for assessing content validity [6]

For multidimensional PROMs, i.e. PROMs that contain multiple subscales, we evaluated each subscale separately. We classified the PROM (subscales) according to our conceptual model (Fig. 1) and rated the relevance and comprehensiveness for measuring the specific concept that the PROM (subscale) was classified into. All ratings in all steps were performed by two reviewers independently. When assessing the quality of the included studies (step 1 and 2) at least one reviewer had expertise in PROM development and evaluation. When assessing the quality of the PROMs (step 3) both reviewers had expertise in PROM development and evaluation. When giving our own ratings of the content of the PROM (step 3a_3) one reviewer had expertise in PROM development and validation, and one reviewer was a clinician with experience in treating people with diabetes. Differences were discussed until consensus was reached.

## Results

### Literature Search

**Fig. 2:**
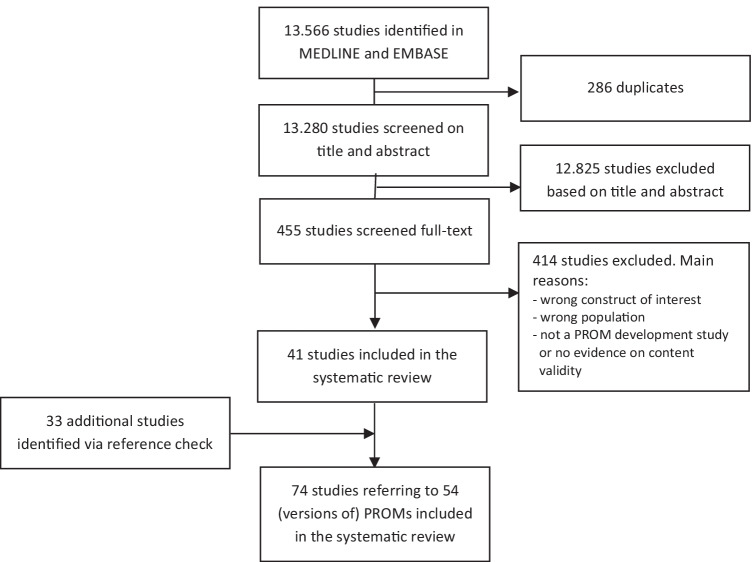
Flow chart of the search strategy

A flow chart of the abstract and article selection is presented in Fig. 2. A total of 13.280 unique abstracts were found, of which 41 articles were included: 23 articles on PROM development and 19 on content validity. Based on reference checking, 24 additional articles on PROM development were identified and nine on content validity, leading to a total of 74 included articles; 46 articles on PROM development, and 28 on content validity.

### PROMs

In total, 54 (different versions of) PROMs were included, containing a total of 150 subscales related to (aspects of) HRQL (full names of the PROMs are listed in Appendix 3). We found 23 (subscales of) PROMs measuring diabetes-specific symptoms, six (subscales of) PROMs measuring energy/fatigue, 32 (subscales of) PROMs measuring distress, 21 (subscales of) PROMs measuring anxiety, three (subscales of) PROMs measuring depressive symptoms, ten (subscales of) PROMs measuring physical function, two PROM subscales measuring sexual function, 11 (subscales of) PROMs measuring emotional function, 24 (subscales of) PROMs measuring social function, and 22 (subscales of) PROMs measuring overall self-rated health. The number of items varied from 1 to 38 per subscale, most scales contained less than 10 items.

#### Step 1: Quality of PROM Development Studies

Details of the populations involved in the PROM development studies are provided in Appendix 4. All ratings of the quality of the PROM development are provided in Appendix 5. For only 24 of 54 (versions of) PROMs (44%), a clear definition of the construct to be measured was provided. Only 27 out of 54 PROMs (50%) were developed with input from people with type 2 diabetes. Twenty-six (48%) PROMs were pilot tested. The total PROM development was rated as inadequate for 46 out of 54 (85%) PROMs and doubtful for seven PROMs (the DD Core [28], DFS [29], DFS-SF [30], DSSI [31], IWADL [32], PRO-DM-Thai [33], and QOLID [34]) (full names and details of the PROMs can be found in Appendix 3). Only one PROM, the Diabetes Questionnaire [35], received an adequate rating for the PROM development.

#### Step 2: Quality of Content Validity Studies

Details of the populations involved in the content validity studies are provided in Appendix 6. All ratings of the quality of the content validity studies can be found in Appendix 7. Twenty-five studies evaluated at least one aspect of content validity (mostly comprehensibility) of 14 PROMs. Most studies were of doubtful quality.

#### Step 3: Quality of the PROMs

We were not able to give a reviewer rating for the quality of five PROMs (diabetes-39 short form 22 items [36], HSM [37], IRD-QOL [38], LQD [39], and QSD [40]), since we did not acquire full-text copies for them even after contacting a large number of authors that used them or developed them (Appendix 3).

Summarizing all evidence per PROM (subscale), only 41 out of 150 PROM subscales (27%) were rated as having sufficient relevance, comprehensiveness, and comprehensibility. PROMs with sufficient content validity are presented in green in Table 3. We found 66 out of 150 PROM subscales (44%) with evidence for insufficient relevance, comprehensiveness, or comprehensibility. The quality of the evidence was mostly low to very low for all PROMs. For each aspect of HRQL (Fig. 1), we identified one to three (subscales of) PROMs with sufficient relevance, comprehensiveness, and comprehensibility, except for depressive symptoms, for which we found no PROM (subscale) with sufficient content validity. Below, we summarize per aspect of HRQL which (subscales of) PROMs were rated to have the best content validity. We also summarize the quality of the evidence, indicating how confident we are that the ratings are trustworthy.

**Table 3:**
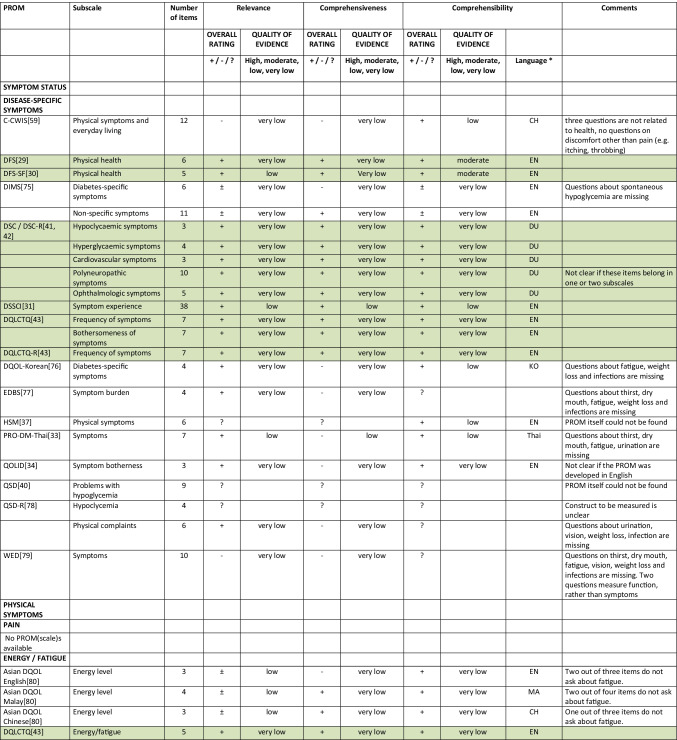

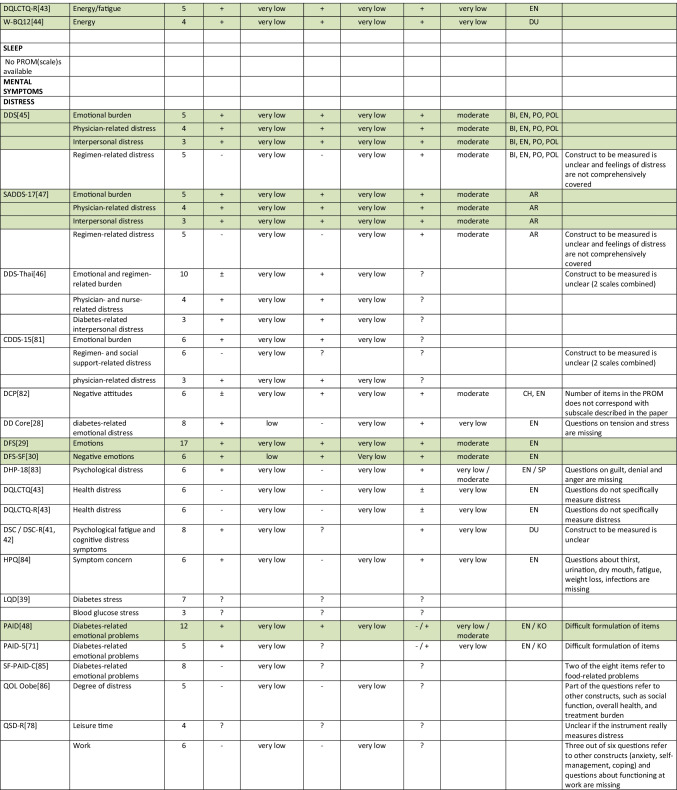

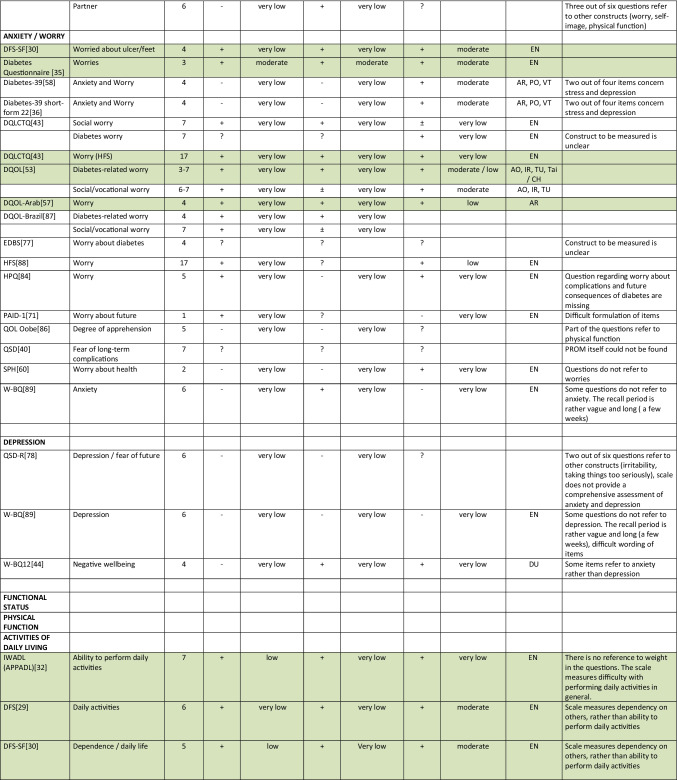

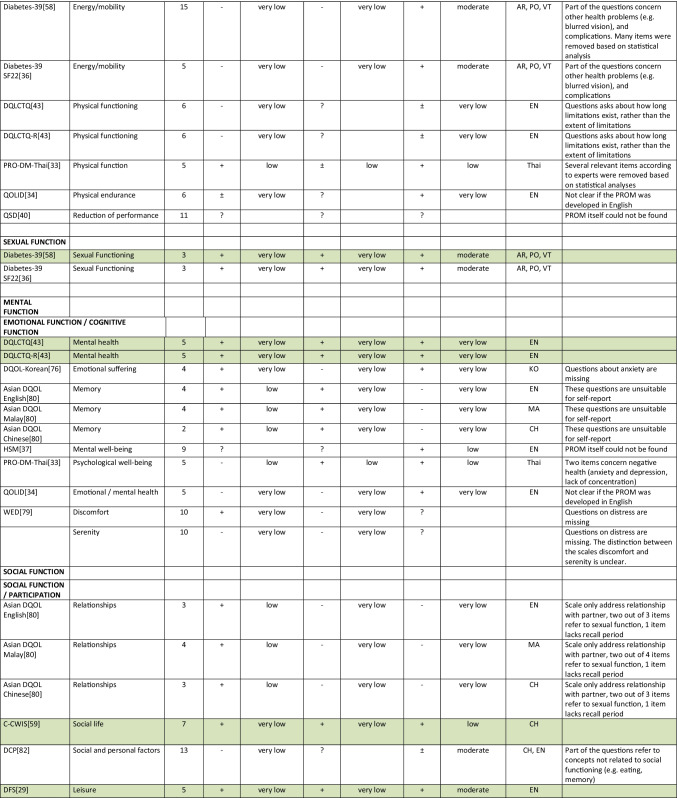

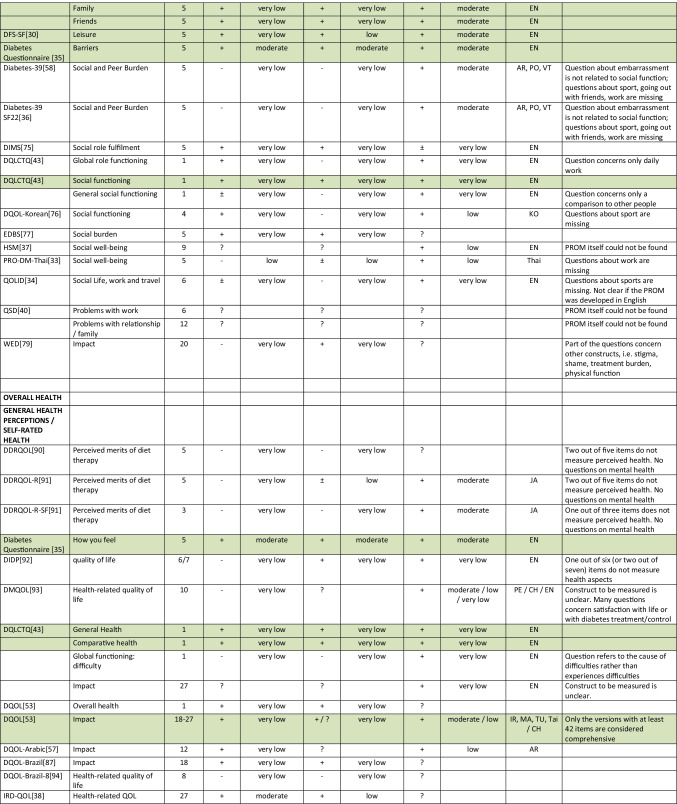

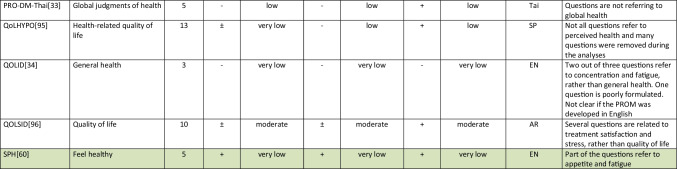
Content validity (relevance, comprehensiveness, comprehensibility) of disease-specific patient-reported health outcome measures developed for patients with type 2 diabetes mellitus (PROMs with positive ratings for content validity are presented in green)

^*^Language in which comprehensibility was rated. *AR*, Arabic; *BI*, Bahasa Indonesia; *CH*, Chinese; *DA*, Danish; *DU*, Dutch; *EN*, English; *IR*, Iranian; *JA*, Japanese; *KO*, Korean; *MA*, Malay; *PE*, Persian; *PO*, Portuguese; *POL*, Polish; *SP*, Spanish; *Tai*, Taiwanese; *Thai*, Thai; *TU*, Turkish; *VT*, Vietnamese

For measuring *diabetes-specific symptoms,* we found sufficient content validity (relevance, comprehensiveness, and comprehensibility) of the DSSCI [31], five subscales of the DSC/DSC-R [41, 42], and two subscales of the DQLCTQ/ DQLCTQ-R [43], but the quality of the evidence was low to very low. For measuring diabetes foot ulcer-specific symptoms, we found sufficient content validity for one subscale of the DFS/DFS-SF, with very low-to-moderate evidence [29, 30].

For measuring *energy/fatigue*, we found sufficient content validity of the DQLCTQ/DQLCTQ-R subscale energy/fatigue [43] and the W-BQ12 subscale energy [44], with very low-quality evidence.

For measuring *distress*, we found sufficient content validity of the DD Core [28], three subscales of the DDS [45, 46], three subscales of the SADDS-17 [47], one subscale of the DFS [29], one of the DFS-SF [30], and the PAID [48]. The quality of the evidence was very low to low for relevance and comprehensiveness and very low to moderate for comprehensibility across languages.

For measuring *anxiety*, we found sufficient content validity of the worry subscale of the Diabetes Questionnaire [35], based on moderate quality evidence, and sufficient content validity of the DFS-SF [30], DQLCTQ [43], DQOL [49–56], and DQOL-Arab [57], based on very low to low-quality evidence.

For measuring *physical function*, we found sufficient content validity of the IWADL [32], DFS [29], and DFS-SF [30], but based on low to very low-quality evidence, with the exception of moderate quality evidence for the comprehensibility of the DFS/DFS-SF.

For measuring *sexual function,*we found sufficient content validity of the Diabetes-39 [58]. The quality of the evidence was very low for relevance and comprehensiveness and moderate for comprehensibility.

For measuring *emotional function*, we found sufficient content validity of the mental health subscale of the DQLCTQ/DQLCT-R [43], with very low-quality evidence.

For measuring *social function*, we found sufficient content validity of the social life subscale or the C-CWIS [59], the barriers subscale of the Diabetes Questionnaire[35], four subscales of the DFS/DFS-SF [29, 30], and a single item of the DQLCTQ [43]. The quality of the evidence was moderate for the Diabetes Questionnaire, very low to moderate for the DFS/DFS-SF, very low to low for the C-CWIS, and very low for the DQLCTQ.

Finally, for measuring overall *self-rated health*, we found sufficient content validity of the how you feel subscale of the Diabetes Questionnaire[35], the impact subscale of the 42 + item versions of the DQOL [49–56], the subscale feel healthy of the SPH [60], and two items of the DQLCTQ that were developed to be used as single items [43]. The quality of the evidence was moderate for the Diabetes Questionnaire, very low to moderate for the DQOL, and very low for the SPH and DQLCTQ.

## Discussion

We systematically evaluated the content validity of PROMs specifically developed to measure (aspects of) HRQOL in people with type 2 diabetes. We found evidence for sufficient content validity for only 41 out of the 150 (27%) included PROM subscales. For each aspect of HRQL, we identified one to 11 (subscales of) PROMs with sufficient content validity, except for depressive symptoms, for which we found no PROM (subscale) with sufficient content validity. However, the quality of evidence was generally low to very low. The highest quality evidence was found for the Diabetes Questionnaire subscales worries (measuring anxiety), barriers (measuring social function), and how you feel (measuring general health perceptions), for the DSSCI measuring symptom experience, and the IWADL measuring the ability to participate in daily activities.

Our results and conclusions differ from previous reviews [9–22] because these reviews did not provide a comprehensive overview of content validity, did not take the quality of the PROM development into account, or did not consider evidence for relevance, comprehensiveness, and comprehensibility separately. Striking is that some of the PROMs with the best evidence for content validity based on our review (Diabetes Questionnaire, DFS, and IWADL) were not included in the most recent review, by Wee et al. [22], indicating that their review was likely incomplete.

We found moderate evidence for the comprehensibility of many PROMs, indicating that the questions seem well understood by people with type 2 diabetes across different languages. However, the quality of the evidence for relevance and comprehensiveness of most PROMS was very low. More high-quality research is warranted to determine if these PROMS measure the most relevant aspects of HRQOL for people with type 2 diabetes.

The quality of the PROM development studies was considered inadequate for 85% of the included PROMs. Only half of the PROMs were developed with (some) input from people with type 2 diabetes. This is a major limitation because it is well-known that patients and healthcare professionals may have different opinions about important outcomes to measure. Also, many PROMs are modified versions of previously developed PROMs. Items were often removed based on statistical analyses without addressing the relevance of these items for people with type 2 diabetes. Also, the decision to add new items was often not discussed with people with type 2 diabetes. Furthermore, for more than half of the PROMs, it was unclearly described what the PROM (subscales) exactly aimed to measure. Undefined names are used, such as “physical health”, “emotional burden”, “dependence”, “impact”, or “how you feel”. The content of the (subscales of) PROMs is often very different (even though they claim to measure the same construct) and a rationale for the questions within scales is not provided. If what is being measured is unclear or not based on what is most relevant for the target population, this may affect other measurement properties, such as responsiveness. Furthermore, it will impede the identification of the best PROM for a specific context of use, it will hamper interpretation and comparison of PROM results in studies, and it will limit the usefulness of PROMs in clinical practice.

Another striking finding of this review is that many PROMs that claim to measure (aspects of) HRQOL measure in fact (partly) other things, such as contextual factors or patient experiences (Appendix 3). Examples of contextual factors are behaviour (diet adherence, self-management), attitudes, stigma, support, or financial worries [2]. These are important factors that influence HRQOL, but they are not aspects of HRQOL. Examples of patient experiences are treatment satisfaction, treatment burden or barriers, and doctor–patient relationship. These are patient experience measures (PREMs), not PROMs [61]. It should be noted, however, that many of the included PROMs were developed many years ago, when the methodology of PROM development and validation was not yet as strongly developed as it is today.

The large number of available (versions of) PROMs (and subscales) and the variety in content being measured with these PROMs suggests lack of consensus on which aspects of HRQOL are most relevant to measure in people with type 2 diabetes and how to measure them. Recent initiatives towards standardization of outcomes may improve this situation. Harman et al. recently established international consensus among a large group of people with type 2 diabetes and healthcare providers on the most important outcomes to be measured in clinical trials in people with type 2 diabetes. They identified global quality of life and activities of daily living as two core patient-reported outcomes [62]. We did not include PROMs for measuring global quality of life in our review, but we found sufficient content validity of the IWADL[32] for measuring activities of daily living. A second initiative, the International Consortium for Health Outcomes Measurement (ICHOM), developed a standard set of outcomes to be measured in all type 1 and type 2 diabetes patients in clinical practice. They included psychological well-being, depression, and distress as core outcomes and recommend the WHO5, the PHQ9, and the PAID for measuring these outcomes, respectively [63]. The WHO5 and PHQ9 were not included in this review because they are not diabetes-specific. A recent review of the WHO5 concluded that this PROM has adequate validity [64]. It should be noted that the WHO5 is often used to measure depression but actually measures well-being. Another systematic review identified good measurement properties of the PHQ9, although evidence on the content validity for people with type 2 diabetes is lacking [13]. We found sufficient content validity for the PAID [48] to measure distress, although with very low evidence.

Unfortunately, these two sets do not contain the same outcomes, while there is no justification why the most important outcomes to measure in clinical trials would be different from those in clinical practice. Skovlund et al. reviewed recent evidence and key opportunities and challenges for the clinical use of PROMs to support person-centred diabetes care. They recommended most of the above mentioned outcomes (quality of life, self-reported health, depression, anxiety, and distress) to measure in routine diabetes care [1]. Finally, there is increasing evidence that across adults having different kind of diseases, the same patient-reported health outcomes are important, such as fatigue, sleep disturbances, anxiety, depression, physical function, and the ability to participate in social roles and activities [65–67]. All these studies provide important input for what to measure routinely in people with type 2 diabetes.

### Recommendations for Further Research

This review shows the need for more high-quality content validity studies on diabetes-specific HRQL PROMs. Furthermore, the evidence on other measurement properties of those PROMs with sufficient content validity should be summarized in a next review, or evidence from previous reviews [9–21] should be updated. Wee et al. recently performed such a review [22], but their review was likely incomplete.

In addition, we recommend to consider the Patient-Reported Outcomes Measurement Information System (PROMIS) for future validation studies in people with type 2 diabetes [67]. PROMIS is a set of generic, high-quality, and efficient PROMs, based on modern psychometric methods (item response theory)[68] that measure relevant outcomes such as fatigue, sleep disturbances, anxiety, depression, physical function, and the ability to participate in social roles and activities. PROMIS measures have been extensively validated and are increasingly being used across different (patient) populations [69]. PROMIS measures are especially suitable for people with multiple medical conditions who would otherwise need to complete multiple PROMs for different health care providers. PROMIS measures are already used in routine care for people with diabetes [70] but as far as we know have not yet been validated in people with diabetes.

### Recommendations for the Use of PROMs in Research and Clinical Practice

We recommend that researchers and clinicians first consider carefully which aspects of HRQOL are most relevant to measure in their specific context. We recommend to involve people with type 2 diabetes in this selection process. We also recommend to consider outcomes that have shown to be relevant for many (patient) populations, such as fatigue, sleep disturbances, anxiety, depression, physical function, and the ability to participate in social roles and activities. We recommend to use (subscales of) PROMs with sufficient content validity (presented in green in Table 3), such as the DSSCI for measuring disease-specific symptoms, the Diabetes Questionnaire subscales for measuring worries and general health perceptions, and the IWADL measuring the ability to participate in daily activities. As an alternative, high-quality generic PROMs, such as the WHO5, PHQ9, and PROMIS, may be considered. We recommend not to use the 61 PROM subscales identified in this review with evidence for either insufficient relevance, insufficient comprehensiveness, or insufficient comprehensibility.

### Limitations

This review has some limitations. First, we identified PROMs based on screening studies on PROM development or content validity. However, additional (versions of) PROMs may have been developed, for example, based on factor analysis, published in papers on other measurement properties. Not all of these papers were identified through our screening approach which means that this review may not include all existing (versions of) PROMs. However, PROMs based on statistic methods only would not be rated as having sufficient content validity, so we are quite confident that we did not miss PROMs with good content validity.

Second, we could not rate five PROMs because we were unable to find full copies of the PROMs, it was not always possible to distinguish between different versions of a PROM, and it was sometimes difficult to distinguish PROM development studies from content validity studies. This, as well as poor reporting of development and validation studies, may have led to underestimation of some of our ratings. Third, we found many papers by reference checking, which may indicate lack of comprehensiveness of the original search strategy. However, we were not able to identify additional search terms that would have identified these papers. It is likely that papers were not included in the search due to poor reporting of content validity details in the abstracts.

The strengths of our review were the extensive search strategy, with more than 13,000 papers screened and extensive reference checking, and the detailed and transparent assessment of all aspects of content validity, using the consensus-based COSMIN methodology [6].

## Conclusion

We found 54 (different versions of) PROMs, containing a total of 150 subscales measuring (aspects of) HRQL in people with type 2 diabetes. Only 41 of these 150 subscales (27%) were rated as having sufficient content validity. For each aspect of HRQL, we found one to 11 (subscales of) PROMs with sufficient relevance, comprehensiveness, and comprehensibility, except for depressive symptoms. The quality of the evidence was, however, mostly very low. In order to help clinicians and researchers to select those PROMs that are most suited for the intended purpose, future reviews should evaluate other measurement properties of those PROMs with sufficient content validity. Additionally, the use of generic PROMs in people with diabetes needs more study.

## Supplementary Information

Below is the link to the electronic supplementary material.

Supplementary file1 (DOCX 16 KB)

## Data Availability

All data extracted or analysed during this study are included in this published article and its supplementary information files.
