# Supplementary material for: Content Validity of Patient-Reported Outcome Measures Developed for Assessing Health-Related Quality of Life in People with Type 2 Diabetes Mellitus: a Systematic Review
Source: Curr Diab Rep. 2022 Jul 11;22(9):405–21. doi: 10.1007/s11892-022-01482-z (PMC9355936; doi:10.1007/s11892-022-01482-z)
Supplement: Supplementary file 1 — Supplementary file1 (DOCX 16 KB) [file 11892_2022_1482_MOESM1_ESM.docx]

**Supplementary materials associated with this paper:**

**Content validity of patient-reported outcome measures developed for assessing health-related quality of life in people with type 2 diabetes mellitus – a systematic review**

Caroline B. Terwee, Petra J.M. Elders, Marlous Langendoen-Gort, Ellen B.M. Elsman, Cecilia A.C. Prinsen, Amber A. van der Heijden, Maartje de Wit, Joline W.J. Beulens, Lidwine B. Mokkink, Femke Rutters

**Appendix 1. Definitions of measurement properties according to the COSMIN taxonomy.[5]**

| Term |  |  | Definition |
| --- | --- | --- | --- |
| Domain | Measurement property | Aspect of a measurement property |  |
| Reliability |  |  | The degree to which the measurement is free from measurement error |
| Reliability (extended definition) |  |  | The extent to which scores for patients who have not changed are the same for repeated measurement under several conditions: e.g. using different sets of items from the same health related-patient reported outcomes (HR-PRO) (internal consistency); over time (test-retest); by different persons on the same occasion (inter-rater); or by the same persons (i.e. raters or responders) on different occasions (intra-rater) |
|  | Internal consistency |  | The degree of the interrelatedness among the items |
|  | Reliability |  | The proportion of the total variance in the measurements which is due to ‘true’^†^ differences between patients |
|  | Measurement error |  | The systematic and random error of a patient’s score that is not attributed to true changes in the construct to be measured |
| Validity |  |  | The degree to which an HR-PRO instrument truly measures the construct(s) it purports to measure |
|  | Content validity |  | The degree to which the content of an HR-PRO instrument is an adequate reflection of the construct to be measured |
|  |  | Face validity | The degree to which (the items of) an HR-PRO instrument indeed looks as though they are an adequate reflection of the construct to be measured |
|  | Construct validity |  | The degree to which the scores of an HR-PRO instrument are consistent with hypotheses *(for instance with regard to internal relationships, relationships to scores of other instruments, or differences between relevant groups)* based on the assumption that the HR-PRO instrument validly measures the construct to be measured |
|  |  | Structural validity | The degree to which the scores of an HR-PRO instrument are an adequate reflection of the dimensionality of the construct to be measured |
|  |  | Hypotheses testing | Idem construct validity |
|  |  | Cross-cultural validity | The degree to which the performance of the items on a translated or culturally adapted HR-PRO instrument are an adequate reflection of the performance of the items of the original version of the HR-PRO instrument |
|  | Criterion validity |  | The degree to which the scores of an HR-PRO instrument are an adequate reflection of a ‘gold standard’ |
| Responsiveness |  |  | The ability of an HR-PRO instrument to detect change over time in the construct to be measured |
|  | Responsiveness |  | Idem responsiveness |

**Appendix 2. Search strategy**

**PUBMED search April 29, 2019**

#1 Diabetes type 2

((Diabet*[tiab] AND (("non insulin"[tiab] AND depend*[tiab]) OR ("noninsulin"[tiab] AND depend*[tiab]) OR “type 2”[tiab] OR “type II” [tiab])) OR iddm[tiab] OR niddm[tiab] OR “glucose intolerance”[tiab] OR “insulin resistant”[tiab] OR “insulin resistance”[tiab])

#2 Modified filter for studies on measurement properties*

~~instrumentation[sh] OR methods[sh] OR~~ "Validation Studies"[pt] ~~OR "Comparative Study"[pt]~~ OR "psychometrics"[MeSH] OR psychometr*[tiab] OR clinimetr*[tw] OR clinometr*[tw] OR "outcome assessment (health care)"[MeSH] OR "outcome assessment"[tiab] OR "outcome measure*"[tw] OR "observer variation"[MeSH] OR "observer variation"[tiab] ~~OR "Health Status Indicators"[Mesh]~~ OR "reproducibility of results"[MeSH] OR reproducib*[tiab] OR "discriminant analysis"[MeSH] OR reliab*[tiab] OR unreliab*[tiab] OR valid*[tiab] OR "coefficient of variation"[tiab] ~~OR coefficient[tiab]~~ OR homogeneity[tiab] OR homogeneous[tiab] OR "internal consistency"[tiab] OR (cronbach*[tiab] AND (alpha[tiab] OR alphas[tiab])) OR (item[tiab] AND (correlation*[tiab] OR selection*[tiab] OR reduction*[tiab])) OR agreement[tw] OR precision[tw] OR imprecision[tw] OR "precise values"[tw] OR test-retest[tiab] OR (test[tiab] AND retest[tiab]) OR (reliab*[tiab] AND (test[tiab] OR retest[tiab])) OR stability[tiab] OR interrater[tiab] OR inter-rater[tiab] OR intrarater[tiab] OR intra-rater[tiab] OR intertester[tiab] OR inter-tester[tiab] OR intratester[tiab] OR intra-tester[tiab] OR interobserver[tiab] OR inter-observer[tiab] OR intraobserver[tiab] OR intra-observer[tiab] OR intertechnician[tiab] OR inter-technician[tiab] OR intratechnician[tiab] OR intra-technician[tiab] OR interexaminer[tiab] OR inter-examiner[tiab] OR intraexaminer[tiab] OR intra-examiner[tiab] OR interassay[tiab] OR inter-assay[tiab] OR intraassay[tiab] OR intra-assay[tiab] OR interindividual[tiab] OR inter-individual[tiab] OR intraindividual[tiab] OR intra-individual[tiab] OR interparticipant[tiab] OR inter-participant[tiab] OR intraparticipant[tiab] OR intra-participant[tiab] OR kappa[tiab] OR kappa's[tiab] OR kappas[tiab] OR repeatab*[tw] OR ((replicab*[tw] OR repeated[tw]) AND (measure[tw] OR measures[tw] OR findings[tw] ~~OR result[tw] OR results[tw]~~ OR test[tw] OR tests[tw])) OR generaliza*[tiab] OR generalisa*[tiab] OR concordance[tiab] OR (intraclass[tiab] AND correlation*[tiab]) OR discriminative[tiab] OR "known group"[tiab] OR "factor analysis"[tiab] OR "factor analyses"[tiab] OR "factor structure"[tiab] OR "factor structures"[tiab] ~~OR dimension*[tiab]~~ OR subscale*[tiab] OR (multitrait[tiab] AND scaling[tiab] AND (analysis[tiab] OR analyses[tiab])) OR "item discriminant"[tiab] OR "interscale correlation*"[tiab] OR error[tiab] OR errors[tiab] OR "individual variability"[tiab] OR "interval variability"[tiab] OR "rate variability"[tiab] OR (variability[tiab] AND (analysis[tiab] OR values[tiab])) ~~OR (uncertainty[tiab] AND (measurement[tiab] OR measuring[tiab])~~) OR "standard error of measurement"[tiab] ~~OR sensitiv*[tiab]~~ OR responsive*[tiab] OR (limit[tiab] AND detection[tiab]) OR "minimal detectable concentration"[tiab] OR interpretab*[tiab] OR ((minimal[tiab] OR minimally[tiab] OR clinical[tiab] OR clinically[tiab]) AND (important[tiab] ~~OR significant[tiab]~~ OR detectable[tiab]) AND (change[tiab] OR difference[tiab])) OR (small*[tiab] AND (real[tiab] OR detectable[tiab]) AND (change[tiab] OR difference[tiab])) OR "meaningful change"[tiab] OR "ceiling effect"[tiab] OR "floor effect"[tiab] OR "Item response model"[tiab] OR IRT[tiab] OR Rasch[tiab] OR "Differential item functioning"[tiab] OR DIF[tiab] OR "computer adaptive testing"[tiab] OR "item bank"[tiab] OR "cross-cultural equivalence"[tiab]

#3 PROM filter (developed by the University of Oxford, see www.comin.nl)

(HR-PRO[tiab] OR HRPRO[tiab] OR HRQL[tiab] OR HRQoL[tiab] OR QL[tiab] OR QoL[tiab] OR quality of life[tw] OR life quality[tw] OR health index*[tiab] OR health indices[tiab] OR health profile*[tiab] OR health status[tw] OR ((patient[tiab] OR self[tiab] OR child[tiab] OR parent[tiab] OR carer[tiab] OR proxy[tiab]) AND ((report[tiab] OR reported[tiab] OR reporting[tiab]) OR (rated[tiab] OR rating[tiab] OR ratings[tiab]) OR based[tiab] OR (assessed[tiab] OR assessment[tiab] OR assessments[tiab]))) OR ((disability[tiab] OR function[tiab] OR functional[tiab] OR functions[tiab] OR subjective[tiab] OR utility[tiab] OR utilities[tiab] OR wellbeing[tiab] OR well being[tiab]) AND (index[tiab] OR indices[tiab] OR instrument[tiab] OR instruments[tiab] OR measure[tiab] OR measures[tiab] OR questionnaire[tiab] OR questionnaires[tiab] OR profile[tiab] OR profiles[tiab] OR scale[tiab] OR scales[tiab] OR score[tiab] OR scores[tiab] OR status[tiab] OR survey[tiab] OR surveys[tiab])))

(#1 AND #2 AND #3) NOT ("addresses"[Publication Type] OR "biography"[Publication Type] OR "case reports"[Publication Type] OR "comment"[Publication Type] OR "directory"[Publication Type] OR "editorial"[Publication Type] OR "festschrift"[Publication Type] OR "interview"[Publication Type] OR "lectures"[Publication Type] OR "legal cases"[Publication Type] OR "legislation"[Publication Type] OR "letter"[Publication Type] OR "news"[Publication Type] OR "newspaper article"[Publication Type] OR "patient education handout"[Publication Type] OR "popular works"[Publication Type] OR "congresses"[Publication Type] OR "consensus development conference"[Publication Type] OR "consensus development conference, nih"[Publication Type] OR "practice guideline"[Publication Type]) NOT ("animals"[MeSH Terms] NOT "humans"[MeSH Terms])

**EMBASE search April 29, 2019**

#1 diabetes type 2

(Diabet*:ti,ab AND (('non insulin':ti,ab AND depend*:ti,ab) OR (noninsulin:ti,ab AND depend*:ti,ab) OR 'type 2':ti,ab OR 'type II':ti,ab)) OR iddm:ti,ab OR niddm:ti,ab OR 'glucose intolerance':ti,ab OR 'insulin resistant':ti,ab OR 'insulin resistance':ti,ab

#2 Modified filter for studies on measurement properties^1^

~~'intermethod comparison'/exp OR~~ 'data collection method'/exp OR 'validation study'/exp OR 'feasibility study'/exp OR 'pilot study'/exp OR 'psychometry'/exp OR 'reproducibility'/exp OR reproducib*:ab,ti OR 'audit':ab,ti OR psychometr*:ab,ti OR clinimetr*:ab,ti OR clinometr*:ab,ti OR 'observer variation'/exp OR 'observer variation':ab,ti OR 'discriminant analysis'/exp OR 'validity'/exp OR reliab*:ab,ti OR valid*:ab,ti ~~OR 'coefficient':ab,ti~~ OR 'internal consistency':ab,ti OR (cronbach*:ab,ti AND ('alpha':ab,ti OR 'alphas':ab,ti)) OR 'item correlation':ab,ti OR 'item correlations':ab,ti OR 'item selection':ab,ti OR 'item selections':ab,ti OR 'item reduction':ab,ti OR 'item reductions':ab,ti OR 'agreement':ab,ti OR 'precision':ab,ti OR 'imprecision':ab,ti OR 'precise values':ab,ti OR 'test-retest':ab,ti OR ('test':ab,ti AND 'retest':ab,ti) OR (reliab*:ab,ti AND ('test':ab,ti OR 'retest':ab,ti)) OR 'stability':ab,ti OR 'interrater':ab,ti OR 'inter-rater':ab,ti OR 'intrarater':ab,ti OR 'intra-rater':ab,ti OR 'intertester':ab,ti OR 'inter-tester':ab,ti OR 'intratester':ab,ti OR 'intra-tester':ab,ti OR 'interobeserver':ab,ti OR 'inter-observer':ab,ti OR 'intraobserver':ab,ti OR 'intra-observer':ab,ti OR 'intertechnician':ab,ti OR 'inter-technician':ab,ti OR 'intratechnician':ab,ti OR 'intra-technician':ab,ti OR 'interexaminer':ab,ti OR 'inter-examiner':ab,ti OR 'intraexaminer':ab,ti OR 'intra-examiner':ab,ti OR 'interassay':ab,ti OR 'inter-assay':ab,ti OR 'intraassay':ab,ti OR 'intra-assay':ab,ti OR 'interindividual':ab,ti OR 'inter-individual':ab,ti OR 'intraindividual':ab,ti OR 'intra-individual':ab,ti OR 'interparticipant':ab,ti OR 'inter-participant':ab,ti OR 'intraparticipant':ab,ti OR 'intra-participant':ab,ti OR 'kappa':ab,ti OR 'kappas':ab,ti OR 'coefficient of variation':ab,ti OR repeatab*:ab,ti OR (replicab*:ab,ti OR 'repeated':ab,ti AND ('measure':ab,ti OR 'measures':ab,ti OR 'findings':ab,ti ~~OR 'result':ab,ti OR 'results':ab,ti~~ OR 'test':ab,ti OR 'tests':ab,ti)) OR generaliza*:ab,ti OR generalisa*:ab,ti OR 'concordance':ab,ti OR ('intraclass':ab,ti AND correlation*:ab,ti) OR 'discriminative':ab,ti OR 'known group':ab,ti OR 'factor analysis':ab,ti OR 'factor analyses':ab,ti OR 'factor structure':ab,ti OR 'factor structures':ab,ti OR 'dimensionality':ab,ti OR subscale*:ab,ti OR 'multitrait scaling analysis':ab,ti OR 'multitrait scaling analyses':ab,ti OR 'item discriminant':ab,ti OR 'interscale correlation':ab,ti OR 'interscale correlations':ab,ti OR ('error':ab,ti OR 'errors':ab,ti AND (measure*:ab,ti OR correlat*:ab,ti OR evaluat*:ab,ti OR 'accuracy':ab,ti OR 'accurate':ab,ti OR 'precision':ab,ti OR 'mean':ab,ti)) OR 'individual variability':ab,ti OR 'interval variability':ab,ti OR 'rate variability':ab,ti OR 'variability analysis':ab,ti OR 'standard error of measurement':ab,ti ~~OR sensitiv*:ab,ti~~ OR responsive*:ab,ti OR ('limit':ab,ti AND 'detection':ab,ti) OR 'minimal detectable concentration':ab,ti OR interpretab*:ab,ti OR (small*:ab,ti AND ('real':ab,ti OR 'detectable':ab,ti) AND ('change':ab,ti OR 'difference':ab,ti)) OR 'meaningful change':ab,ti OR 'minimal important change':ab,ti OR 'minimal important difference':ab,ti OR 'minimally important change':ab,ti OR 'minimally important difference':ab,ti OR 'minimal detectable change':ab,ti OR 'minimal detectable difference':ab,ti OR 'minimally detectable change':ab,ti OR 'minimally detectable difference':ab,ti OR 'minimal real change':ab,ti OR 'minimal real difference':ab,ti OR 'minimally real change':ab,ti OR 'minimally real difference':ab,ti OR 'ceiling effect':ab,ti OR 'floor effect':ab,ti OR 'item response model':ab,ti OR 'irt':ab,ti OR 'rasch':ab,ti OR 'differential item functioning':ab,ti OR 'dif':ab,ti OR 'computer adaptive testing':ab,ti OR 'item bank':ab,ti OR 'cross-cultural equivalence':ab,ti

#3 PROM filter (developed by the University of Oxford, see www.comin.nl)

(HR-PRO:ti,ab OR HRPRO:ti,ab OR HRQL:ti,ab OR HRQoL:ti,ab OR QL:ti,ab OR QoL:ti,ab OR 'quality of life':ti,ab OR 'life quality':ti,ab OR 'health index*':ti,ab OR 'health indices':ti,ab OR 'health profile*':ti,ab OR 'health status':ti,ab OR ((patient:ti,ab OR self:ti,ab OR child:ti,ab OR parent:ti,ab OR carer:ti,ab OR proxy:ti,ab) AND ((report:ti,ab OR reported:ti,ab OR reporting:ti,ab) OR (rated:ti,ab OR rating:ti,ab OR ratings:ti,ab) OR based:ti,ab OR (assessed:ti,ab OR assessment:ti,ab OR assessments:ti,ab))) OR ((disability:ti,ab OR function:ti,ab OR functional:ti,ab OR functions:ti,ab OR subjective:ti,ab OR utility:ti,ab OR utilities:ti,ab OR wellbeing:ti,ab OR 'well being':ti,ab) AND (index:ti,ab OR indices:ti,ab OR instrument:ti,ab OR instruments:ti,ab OR measure:ti,ab OR measures:ti,ab OR questionnaire:ti,ab OR questionnaires:ti,ab OR profile:ti,ab OR profiles:ti,ab OR scale:ti,ab OR scales:ti,ab OR score:ti,ab OR scores:ti,ab OR status:ti,ab OR survey:ti,ab OR surveys:ti,ab)))

#4 publicatie types

#3 AND ('article'/it OR 'article in press'/it OR 'review'/it)

#5 not animals

#4 NOT ([animals]/lim NOT [humans]/lim)

^1^ Modified from Terwee et al. 2009 [97]. The crossed out search terms were left out because these terms, in combination with the search terms for diabetes, yielded too many abstracts to read.

**Appendix 3. Included PROMs (grey subscales were excluded from this review because they do not measure aspects of HRQL)**

| **PROM** | **Full name** | **Target population** | **Construct(s)** | **Subscales (number of items)** |
| --- | --- | --- | --- | --- |
| AsianDQOL Chinese-Mandarin[80] | Asian Diabetes Quality of Life | Diabetes | Diabetes-related quality of life | Financial concerns (6)  Relationship (3)  Memory (2)  Diet and activities (4)  Energy levels (3) |
| AsianDQOL English[80] | Asian Diabetes Quality of Life | Diabetes | Diabetes-related quality of life | Financial concerns (5)  Relationship (3)  Memory (4)  Diet and activities (6)  Energy levels (3) |
| AsianDQOL Malay[80] | Asian Diabetes Quality of Life | Diabetes | Diabetes-related quality of life | Financial concerns (5)  Relationship (4)  Memory (4)  Diet and activities (4)  Energy levels (4) |
| C-CWIS[59] | Chinese Cardiff Wound Impact Schedule | Diabetic foot ulcers | Health-related quality of life | Physical symptoms and everyday living (12)  Social life (7)  Well-being (6) |
| DCP[82] | Diabetes Care Profile | Diabetes | Social and psychological aspects of diabetes and its treatment | Control problems (18)  Social and personal factors (13)  Positive attitude (5)  Negative attitude (6)  Self-care ability (4)  Importance of care (4)  Self-care adherence (4)  Diet adherence (4)  Medical barriers (8)  Exercise barriers (5)  Monitoring barriers (11)  Understanding mgt. practice (10)  Long-term care benefits (5)  Support attitudes (6) |
| DD Core[28] | Diabetes Distress Core items | Type 2 diabetes patients | Diabetes-related emotional distress | DD Core (8) |
| DDRQOL[90] | Diabetes Diet-Related Quality of Life | Type 2 diabetes patients | Diabetes diet-related quality of life | Satisfaction with diet (4)  Burden of diet therapy (8)  Perceived merits of diet therapy (5)  General perception of diet (1)  Restriction of social functions (2)  Vitality (4)  Mental health (5) |
| DDRQOL-R[91] | Diabetes Diet-Related Quality of Life - Revised | Type 2 diabetes patients | Diabetes diet-related quality of life | Satisfaction with diet (4)  Burden of diet therapy (8)  Perceived merits of diet therapy (5) |
| DDRQOL-R short form[91] | Diabetes Diet-Related Quality of Life - Revised | Type 2 diabetes patients | Diabetes diet-related quality of life | Satisfaction with diet (3)  Burden of diet therapy (3)  Perceived merits of diet therapy (3) |
| DDS[45] | Diabetes Distress Scale | Diabetes | Diabetes distress | Emotional burden (5)  Physician-related distress (4)  Regimen-related distress (5)  Interpersonal distress (3) |
| SADDS-17[47] | Diabetes Distress Scale | Diabetes | Diabetes distress | Emotional burden (5)  Physician-related distress (4)  Regimen-related distress (5)  Interpersonal distress (3) |
| DDS Thai[46] | Diabetes Distress Scale Thai version | Diabetes | Diabetes distress | Emotional and regimen-related burden (10)  Physician- and nurse-related distress (4)  Diabetes-related interpersonal distress (3) |
| CDDS-15[81] | Chinese Diabetes Distress Scale | Diabetes | Diabetes distress | Emotional burden (6)  Regimen- and social support-related distress (6)  Physician-related distress (3) |
| DFS[29] | Diabetes Foot Ulcer Scale | Diabetic foot ulcers | Quality of life | Leisure (5)  Physical health (6)  Daily activities (6)  Emotions (17)  Non-compliance (2)  Family (5)  Friends (5)  Positive attitude (5)  Treatment (4)  Satisfaction (1)  Financial (2) |
| DFS-SF[30] | Diabetes Foot Ulcer Scale short form | Diabetic foot ulcers | Quality of life | Leisure (5)  Physical health (5)  Dependence / daily life (5)  Negative emotions (6)  Worried about ulcer/feet (4)  Bothered by ulcer care (4) |
| DHP-18[83] | Diabetes Health Profile | Type 2 diabetes | Diabetes-related Psychological Distress and Behavior | Psychological distress (6)  Barriers to activity (7)  Disinhibited eating (5) |
| Diabetes Questionnaire [35] | Diabetes Questionnaire | Diabetes | Important aspects in life for adult individuals with diabetes | How you feel (5)  Your worries (3)  Your capabilities to manage your diabetes (5)  Barriers (5)  Support from others (3) |
| Diabetes-39[58] | Diabetes-39 | Diabetes | Quality of life | Energy and mobility (15)  Diabetes control (12)  Anxiety and worry (4)  Social and peer burden (5)  Sexual functioning (3) |
| Diabetes-39 short form 22-item[36] * | Diabetes-39 short form | Diabetes | Quality of life | Energy and mobility (5)  Diabetes control (5)  Anxiety and worry (4)  Social and peer burden (5)  Sexual functioning (3) |
| DIDP[92, 98] | DAWN2 Impact of Diabetes Profile | Diabetes | Quality of life | Quality of life (6 or 7) |
| DIMS[75] | Diabetes Impact Measurement Scales | Diabetes | Health Status | Diabetes-specific symptoms (6)  Non-specific symptoms (11)  Well-being (11)  Diabetes-related morale (11)  Social role fulfillment (5) |
| DMQoL[93] | Diabetes-Specific Quality of Life Questionnaire | Diabetes | Health-related quality of life | Health-related quality of life (10) |
| DQLCTQ[43] | Diabetes Quality of Life Clinical Trial Questionnaire | Diabetes | Quality of life | General health (1)  Comparative health (1)  Physical functioning (6)  Global role functioning (1)  Global functioning: difficulty (1)  Social functioning (1)  General social functioning (1)  Energy/fatigue (5)  Health distress (6)  Mental health (5)  Satisfaction (18)  Impact (27)  Social worry (7)  Diabetes worry (7)  Worry (17)  Treatment satisfaction (3)  Treatment flexibility (10)  Social stigma (4)  Frequency of symptoms (7)  Bothersomeness of symptoms (7) |
| DQLCTQ-R[43] | Diabetes Quality of Life Clinical Trial Questionnaire Revised | Diabetes | Quality of life | Physical functioning (6)  Energy/fatigue (5)  Health distress (6)  Mental health (5)  Satisfaction (18)  Treatment satisfaction (3)  Treatment flexibility (10)  Frequency of symptoms (7) |
| DQOL[50, 51, 53] ** | Diabetes Quality of Life | Insulin-dependent diabetes | Quality of life | Overall Health (1)  Diabetes-related worry (4)  Social/vocational worry (7)  Worry (14)  Impact (19-27)  Satisfaction (15-18) |
| DQOL-Arabic[57] | Diabetes Quality of Life | Type 2 diabetes | Quality of life | Worry (4)  Impact (12)  Satisfaction (14) |
| DQOL-Brazil[87] | Diabetes Quality of Life | Diabetes | Quality of life | Diabetes-related worry (4)  Social/vocational worry (7)  Impact (18)  Satisfaction (15) |
| DQOL-Brazil-8[94] | Diabetes Quality of Life | Diabetes | Health-related quality of life | Health-related quality of life (8) |
| D-QOL-Korean[76, 99] | Diabetes-specific Quality of Life scale | Diabetes | Diabetes-specific Quality of Life | Diabetes-specific symptoms (4)  Emotional suffering (4)  Social functioning (4)  Treatment adherence (4) |
| DSC[42] | Diabetes Symptom Checklist | Type 2 diabetes | Symptom severity | Psychological fatigue and cognitive distress symptoms (8)  Polyneuropathic symptoms (10)  Cardiovascular symptoms (3)  Ophthalmologic symptoms (5)  Hypoglycemic symptoms (3)  Hyperglycemic symptoms (4) |
| DSC-R[41] | Diabetes Symptom Checklist - Revised | Type 2 diabetes | Symptom severity | Psychological fatigue and cognitive distress symptoms (8)  Polyneuropathic symptoms (10)  Cardiovascular symptoms (3)  Ophthalmologic symptoms (5)  Hypoglycemic symptoms (3)  Hyperglycemic symptoms (4) |
| DSSCI[31] | Diabetes Symptom Self-Care Inventory | Mexican Americans with diabetes | Diabetes symptoms | Symptom experience (38) |
| EDBS[77] | Elderly Diabetes Burden Scale | Elderly with diabetes | Quality of life | Symptom burden (4)  Worry about diabetes (4)  Social burden (5)  Dietary restrictions (4)  Treatment dissatisfaction (3)  Burden by tablets or insulin (3) |
| HFS[88] | Hypoglycemic Fear Survey | Diabetes | Fear of hypoglycemia | Worry (17)  Behavior (10) |
| HPQ[84] | Hypoglycemia Perspectives Questionnaire | Type 2 diabetes | Hypoglycemia frequency, symptoms,  and impact | Symptom concern (6)  Worry (5)  Compensatory behaviour (5) |
| HSM[37] * | Health Status Measure | Older African-American women with type 2 diabetes | Health status | Physical Symptoms (6)  Mental well-being (9)  Social well-being (9) |
| IRD-QOL[38] * | Iranian Diabetes Quality of Life | Diabetes | Quality of life | Health-related quality of life (27)  General quality of life (13) |
| IWADL (APPADL)[32] | Impact of Weight on Activities of Daily Living questionnaire (Ability to Perform Physical Activities of Daily Living) | Type 2 diabetes | Ability to perform daily physical activities | Ability to perform daily activities (7) |
| LQD[39] * | Quality of Life with Diabetes | Type 2 diabetes | Quality of life | Diabetes satisfaction (7)  Diabetes stress (7)  Blood glucose stress (3) |
| PAID[48] | Problem areas in diabetes | Diabetes | Diabetes-related distress | Diabetes-related emotional problems (12)  Treatment problems (2)  Food-related problems (3)  Social support problems (3) |
| PAID-1[71] | Problem areas in diabetes | Diabetes | Diabetes-related distress | Worry about future (1) |
| PAID-5[71] | Problem areas in diabetes | Diabetes | Diabetes-related distress | Diabetes-related emotional problems (5) |
| SF-PAID-C[85] | Short Form - Problem areas in diabetes - Chinese | Diabetes | Diabetes-related distress | Diabetes-related emotional problems and food-related problems (8) |
| PRO-DM-Thai[33] | Patient-reported outcomes in Thai patients with type 2 diabetes mellitus | Type 2 diabetes | Patient-reported outcomes | Physical function (5)  Symptoms (7)  Psychological well-being (5)  Self-care management (12)  Social well-being (5)  Global judgements of health (5)  Satisfaction with care and flexibility of treatment (5) |
| QoLHYPO[95] | Impact of hypoglycemia on the HRQoL of T2DM patients questionnaire | Type 2 diabetes | Health-related quality of life | Health-related quality of life (13) |
| QOLID[34] | Quality of Life for Indian diabetes Patients | Diabetes | Quality of life | Social life, work and travel (6)  Physical endurance (6)  General health (3)  Treatment satisfaction (4)  Symptom botherness (3)  Financial worries (4)  Emotional/mental health (5)  Diet advise tolerance (3) |
| QOL Oobe[86] | Quality of life questionnaire | Type 2 diabetes | Quality of life | Degree of apprehension (5)  Degree of distress (5)  Degree of satisfaction with life (4)  Degree of satisfaction with treatments (4) |
| QOLSID[96] | Quality of Life Scale for Iraqi DM patients | Type 2 diabetes | Quality of life | Quality of life (10) |
| QSD[40] * | Questionnaire on Stress in Diabetic Patients | Diabetes | Stress | Fear of long-term complications (7)  Dietary restrictions (4)  Problems with hypoglycemia (9)  Difficulties with treatment regimen (10)  Problems with acceptance (15)  Reduction of performance (11)  Problems with work (6)  Strained doctor-patient relationship (5)  Problems with relationship or family (12)  Feeling patronized (6) |
| QSD-R[78] | Questionnaire on Stress in Diabetic Patients - Revised | Diabetes | Stress | Leisure (4)  Depression/fear of future (6)  Hypoglycemia (4)  Treatment regimen/diet (9)  Physical complaints (6)  Work (6)  Partner (6)  Doctor-patient relationship (4) |
| SPH[60] | Self-perception of health | Type 2 diabetes | Self-perception of health | Positive self-feeling (6)  Sociality (6)  Attention seeking (4)  Feel healthy (5)  Worry about health (2)  Dependence (4) |
| W-BQ[89] | Well-being questionnaire | Diabetes | Well-being | Depression (6)  Anxiety (6)  Positive well-being (6) |
| W-BQ12[44] | Well-being questionnaire | Diabetes | Well-being | Negative well-being (4)  Energy (4)  Positive well-being (4) |
| WED[79] | Well-being Enquiry for Diabetics | Diabetes | Diabetes-related quality of life | Symptoms (10)  Discomfort (10)  Serenity (10)  Impact (20) |

* We were unable to find a full copy of the PROM

** Different versions contain different subscales and number of items

**Appendix 4. Study populations involved in PROM development**

| **PROM** | **Language** | **Country** | **Patients** | **Patient input** | **Age (mean, SD / range)** | **Gender (% female)** | **Ethnicity** | **Disease characteristics** | **Professionals** | **Characteristics of professionals** | **Professional input** |  |
| --- | --- | --- | --- | --- | --- | --- | --- | --- | --- | --- | --- | --- |
| Asian DQOL[80] | Chinese-Mandarin | US | 10 | concept elicitation |  |  | Mainly Chinese-educated |  | 10 | Endocrinologists, general practitioners, a public health expert and a diabetic nurse | Commented on structure and relevance |  |
|  | English |  | 30 | concept elicitation | different gender and age groups | | Malay (10), Chinese (10), Indian (10) | T2DM, different duration of DM |  |  |  |  |
|  | Malay |  | 10 | concept elicitation |  |  | Malay (6), Chinese (2), Indian (2) |  |  |  |  |  |
| C-CWIS[59] | Chinese | China | 20 | pilot testing |  |  |  | diabetic foot ulcers | 5 | 2 majored in medicine, 3 majored in nursing | transcultural adjustment |  |
| DCP[82] | English | US | 0 |  |  |  |  |  |  |  |  |  |
| DD Core[28] | English | US | >11 | concept elicitation and pilot testing comprehensibility | different ages, genders, races, education level and diabetes backgrounds | | | |  |  |  |  |
| DDRQOL[90] | Japanese | Japan | 0 |  |  |  |  |  | 5 | 1 diabetologist, 2 diabetes educators, 2 nurses | Face validity assessment |  |
| DDRQOL-R[91] | Japanese | Japan | 0 |  |  |  |  |  | 0 |  |  |  |
| DDRQOL-R short form[91] | Japanese | Japan | 0 |  |  |  |  |  | 0 |  |  |  |
| DDS[45] | English | US | several small groups of patients | pilot testing |  |  |  |  | ? | Patients, diabetes, nurse specialists, dietitians, diabetologists, and diabetes-knowledgeable psychologists | Suggest items for the PROM |  |
| DDS Thai[46] | Thai | Thailand | 0 |  |  |  |  |  | 0 |  |  |  |
| CDDS-15[81] | Chinese | China | 0 |  |  |  |  |  | 0 |  |  |  |
| DFS[29] | English | UK | 10 + 14 + 12 | concept elicitation and pilot testing relevance and comprehensibility | 61 (46-74) | 33-40% |  | diabetic foot ulcers | 0 |  |  |  |
| DFS-SF[30] | English |  | 0 |  |  |  |  |  | 0 |  |  |  |
| DHP-18[83] | English/Danish | UK & Denmark | 0 |  |  |  |  |  | 0 |  |  |  |
| Diabetes Questionnaire [35] | Swedish | Sweden | 29 | concept elicitation | 22-81 | 52% |  | T2DM or T2 DM, duration > 5 year | 1 | chairperson of the national patient organisation | relevance and wording |  |
| Diabetes-39[58] | English | US | ? | concept elicitation |  |  |  | diabetes | ? | physicians, certified diabetes educators, pharmacists | concept elicitation |  |
| Diabetes-39 short form 22-item[36] | English | US | 0 |  |  |  |  |  | 0 |  |  |  |
| DIDP[92] | English | Australia | 7 | face validity and acceptability | |  |  | diabetes | 9 | 3 family members, 6 health care professionals | face validity and acceptability |  |
| DIMS[75] | English | US | 0 |  |  |  |  |  | ? | clinicians, a diabetes nurse, a dietician | concept elicitation |  |
| DMQoL[93] | Chinese | Taiwan | 13 | comprehensibility | different ages (6>65yr) | 46% |  |  | 17 | Panel 1: two pharmacists, two family doctors, and a psychometrician. Panel 2: 4 endocrinologists, 4 family doctors, 2 nurse educators, and 2 pharmacists | review draft PROM |  |
| DQLCTQ[43] | English | US | 30 | rating domains |  |  |  | T1DM (23), T2DM (7) | 11 | clinicians / experts on HRQL research | rating domains / evaluating face validity and content validity of draft PROM |  |
| DQLCTQ-R[43] | English | US | 0 |  |  |  |  |  | 0 |  |  |  |
| DQOL[53] | English | US | ? | review draft PROM for relevance and comprehensibility |  |  |  | IDDM | ? | diabetologists, diabetes nurses | review draft PROM |  |
| DQOL[50, 51] | Chinese | Canada | 10 | discuss issues of concern | 54-75 |  |  | T2DM | ? | endocrinologists | discuss issues of concern |  |
| DQOL-Arabic[57] | Arabic | Jordan | ? | ? |  |  |  | T2DM |  |  |  |  |
| DQOL-Brazil[87] | Portuguese | Brazil | 0 |  |  |  |  |  | 0 |  |  |  |
| DQOL-Brazil-8[94] | Portuguese | Brazil | 0 |  |  |  |  |  | 0 |  |  |  |
| D-QOL-Korean[76] | Korean | Korea | 22 + 20 | concept elicitation, comprehensibility | <30 to >61 | 50% |  | diverse group of T2DM / diabetes | 5 | two physicians, two professors of nursing, and one expert in HRQOL | relevance, comprehensibility |  |
| DSC[42] | Dutch | Netherlands | 0 |  |  |  |  |  | 20 | clinicians, diabetologists, general practitioners, diabetes educators | concept elicitation |  |
| DSC-R[41] | English | UK | 0 |  |  |  |  |  | 0 |  |  |  |
| DSSCI[31] | Spanish | US | 45+16 | concept elicitation, comprehensibility | 52 (11) / 54 (10) | 71% / 75% | Mexican Americans | diverse group of T2DM, disease duration 7 (6) / 10 (9) year | | |  |  |
| EDBS[77] | Japanese | Japan | 0 |  |  |  |  |  | 0 |  |  |  |
| HFS[88] | English | UK | 20 | concept elicitation |  |  |  | T1DM | ? | diabetes health care providers | concept elicitation |  |
| HPQ[84] | English, Greek | US, Cyprus | ? | concept elicitation |  |  |  | T2DM | 0 |  |  |  |
| HSM[37] | English | US | 44 + 10 | concept elicitation, comprehensibility and relevance | 40-75 | 100% | African-American | T2DM > 1 year | 2 | African-American health professionals | relevance and comprehensiveness |  |
| IRD-QOL[38] | Iranian | Iran | 15 | concept elicitation | 53 (19-75) | 47% |  | T1DM (2) and T2DM (13), disease duration 11 (1-25) year | 15 + 15 | 11 nurses, 3 physicians, and 1 dietitian / experts in QOL research | concept elicitation |  |
| IWADL (APPADL)[32] | English | US | 54 + 24 | concept elicitation, comprehensibility |  |  |  | T2DM and BMI of 25–40 kg/m2 | 0 |  |  |  |
| LQD[39] | German | Germany | ? | comprehensibility |  |  |  | several groups of patients with T2DM | 0 |  |  |  |
| PAID[48] | English | US | 25 | comprehensibility |  |  |  | T1DM | 10 | diabetes nurse specialists, dietitians, and diabetologists | | |
| PAID-1[71] | English | multi-national | 0 |  |  |  |  |  | 0 |  |  |  |
| PAID-5[71] | English | multi-national | 0 |  |  |  |  |  | 0 |  |  |  |
| SF-PAID-C[85] | Chinese | Taiwan | 0 |  |  |  |  |  | 0 |  |  |  |
| PRO-DM-Thai[33] | Thai | Thailand | 12 + 15 | concept elicitation / comprehensibility, face validity | 61 (49-70) | 50% |  | T2DM, disease duration 11 (2-20) yr | 9 + 17 | three physicians, two nurses, two pharmacists, and two nutritionists | concept elicitation / relevance, comprehensiveness, comprehensibility |  |
| QoLHYPO[95] | Spanish | Spain | 10 + 18 | concept elicitation / relevance, comprehensibility |  |  |  | T2DM | 4 | clinical experts | concept elicitation |  |
| QOLID[34] | English/Hindi? | India | 20 | concept elicitation, comprehensibility |  |  |  | T2DM | 8 | 4 clinicians and 4 diabetes educators | relevance, comprehensibility |  |
| QOL Oobe[86] | Japanese | Japan | 0 |  |  |  |  |  | 0 |  |  |  |
|  |  |  |  |  |  |  |  |  |  |  |  |  |
| QOLSID[96] | Arabic | Iraq | 0 |  |  |  |  |  | 0 |  |  |  |
| QSD[40] | German | Germany | 76 | concept elicitation |  |  |  | diabetes | 5 | diabetologists | concept elicitation |  |
| QSD-R[78] | German | Germany | 0 |  |  |  |  |  | 0 |  |  |  |
| SPH[60] | English/Hindi? | India | 0 |  |  |  |  |  | ? | social science faculty and diabetologists | concept elicitation |  |
| W-BQ[89] | English | UK | 0 |  |  |  |  |  | 0 |  |  |  |
| W-BQ12[44] | Dutch | Netherlands | 0 |  |  |  |  |  | 0 |  |  |  |
| WED[79] | Italian | Italy | ? | ? |  |  |  | diabetes | ? | diabetologists, psychiatrists, nurses | ? |  |

Empty cells indicate missing information

**Appendix 5. Quality of PROM development (studies of at least doubtful quality presented in green)**

| **PROM** |  | **PROM design** | | | | | | | **Cognitive interview (CI) study^2^** | | | | **Total quality PROM development study** |
| --- | --- | --- | --- | --- | --- | --- | --- | --- | --- | --- | --- | --- | --- |
|  |  | **General design requirements** | | | | | **Concept elicitation^1^** | **Total PROM design** | **General design requirements** | **Comprehen-sibility** | **Comprehen-siveness** | **Total CI study** |  |
|  | **Language in which PROM was developed** | **Clear construct** | **Clear origin of construct** | **Clear target population for which the PROM was developed** | **Clear context of use** | **PROM developed in sample representing the target population** |  |  | **CI study performed in sample representing the target population** |  |  |  |  |
| AsianDQOL[80] | Chinese-Mandarin | V | V | V | D | V | D | D | I |  |  | I | I |
| AsianDQOL[80] | English | V | V | V | D | V | D | D | I |  |  | I | I |
| AsianDQOL[80] | Malay | V | V | V | D | V | D | D | I |  |  | I | I |
| C-CWIS[59] | Chinese | I | D | V | V | I |  | I | A | D |  | D | I |
| DCP[82] | English | I | D | V | V | I |  | I |  |  |  |  | I |
| DD Core[28] | English | V | V | V | V | V | D | D | A | D |  | D | D |
| DDRQOL[90] | Japanese | I | D | V | D | I |  | I |  |  |  |  | I |
| DDRQOL-R[91] | Japanese | I | D | V | V | I |  | I |  |  |  |  | I |
| DDRQOL-R short form[91] | Japanese | I | D | V | V | I |  | I |  |  |  |  | I |
| DDS[45] | English | I | I | V | V | D |  | I | D | ? | ? | D | I |
| DDS Thai[46] | Thai | I | I | V | V | I |  | I |  |  |  |  | I |
| CDDS-15[81] | Chinese | I | I | V | V | D |  | I |  |  |  |  | I |
| DFS[29] | English | V | D | V | V | A | D | D | A | D |  | D | D |
| DFS-SF[30] | English | V | D | V | V | A | D | D | A | D |  | D | D |
| DHP-18[83] | English/Danish | I | D | V | V | D | D | I |  |  |  |  | I |
| Diabetes Questionnaire [35] | Swedish | V | V | V | V | V | A | A | V | A | A | A | A |
| Diabetes-39[58] | English | V | V | V | D | D | D | D |  |  |  |  | I |
| Diabetes-39 short form 22-item[36] | English | V | V | V | D | D | D | D |  |  |  |  | I |
| DIDP[92, 98] | English | V | D | V | V | I |  | I |  |  |  |  | I |
| DIMS[75] | English | V | V | V | V | I |  | I |  |  |  |  | I |
| DMQoL[93] | Chinese | I | I | V | V | I |  | I | D | D |  | D | I |
| DQLCTQ[43] | English | V | V | V | V | D | D | D |  |  |  |  | I |
| DQLCTQ-R[43] | English | V | V | V | V | D | D | D |  |  |  |  | I |
| DQOL[53] | English | I | D | V | V | I |  | I | V | D |  | D | I |
| DQOL[50, 51] | Chinese | I | D | V | V | A | D | I | I |  |  | I | I |
| DQOL-Arabic[57] | Arabic | I | D | V | V | D | D | I | D | D |  | D | I |
| DQOL-Brazil[87] | Portuguese | I | D | V | V | I |  | I |  |  |  |  | I |
| DQOL-Brazil-8[94] | Portuguese | I | D | V | V | I |  | I |  |  |  |  | I |
| D-QOL-Korean[76, 99] | Korean | V | D | V | V | I |  | I | D | D |  | D | I |
| DSC[42] | Dutch | V | D | V | V | I |  | I |  |  |  |  | I |
| DSC-R[41] | English | V | D | V | V | I |  | I |  |  |  |  | I |
| DSSCI[31] | Spanish | V | D | V | D | V | D | D | V | D |  | D | D |
| EDBS[77] | Japanese | V | D | V | D | I |  | I |  |  |  |  | I |
| HFS[88] | English | V | D | I | D | D | D | I | D | ? | ? | D | I |
| HPQ[84] | English, Greek | I | D | V | V | A | D | I | ? | ? | ? | ? | I |
| HSM[37] | English | I | D | V | D | V | A | D | A | D |  | D | I |
| IRD-QOL[38] | Iranian | V | V | V | D | A | D | I | I |  |  | I | I |
| IWADL (APPADL)[32] | English | V | D | V | V | V | D | D | V | ? | ? | D | D |
| LQD[39] | German | I | D | V | D | I |  | I |  |  |  |  | I |
| PAID[48] | English | I | I | V | V | I |  | I | I | I |  | I | I |
| PAID-1[71] | English | I | I | V | V | I |  | I | I | I |  | I | I |
| PAID-5[71] | English | I | I | V | V | I |  | I | I | I |  | I | I |
| SF-PAID-C[85] | Chinese | I | I | V | V | I |  | I |  |  |  |  | I |
| PRO-DM-Thai[33] | Thai | V | D | V | D | D | D | D | A | D |  | D | D |
| QoLHYPO[95] | Spanish | I | D | V | V | V | D | I | V | D |  | D | I |
| QOLID[34] | English/Hindi? | V | D | V | V | A | D | D | D | ? | ? | D | D |
| QOL Oobe[86] | Japanese | I | I | V | V | I |  | I |  |  |  |  | I |
| QOLSID[96] | Arabic | I | D | V | V | I |  | I |  |  |  |  | I |
| QSD[40] | German | I | D | V | D | D | D | I |  |  |  |  | I |
| QSD-R[78] | German | I | D | V | D | I |  | I |  |  |  |  | I |
| SPH[60] | English/Hindi? | I | D | V | D | I |  | I |  |  |  |  | I |
| W-BQ[89] | English | I | D | V | V | I |  | I |  |  |  |  | I |
| W-BQ12[44] | Dutch | I | D | V | V | I |  | I |  |  |  |  | I |
| WED[79] | Italian | V | V | V | V | D | D | D |  |  |  |  | I |

V = very good; A = adequate; D = doubtful; I = inadequate. Empty cells indicate that these aspects were not assessed.

**Appendix 6. Study populations involved in content validity studies**

| **PROM** | **Language** | **Country** | **Setting** | **Patients** | **Age (mean, SD / range)** | **Gender (% female)** | **Disease characteristics** | **Professionals** | **Characteristics of professionals** |
| --- | --- | --- | --- | --- | --- | --- | --- | --- | --- |
| DCP[100] | Chinese | China | Diabetes outpatient clinic | 20 |  |  |  | 5 | 2 endocrinologists, 3 diabetes nurse specialists |
| DDRQOL-R[91] | Japanese | Japan |  | 3 |  |  | diabetes necropathy stage 3 or higher | 62 | 21 specialists from the Japan Diabetes Society, 20 nurses, 21 dieticians / diabetes educators |
| DDRQOL-R short form[91] | Japanese | Japan |  | 0 |  |  |  | 62 | 21 specialists from the Japan Diabetes Society, 20 nurses, 21 dieticians / diabetes educators |
| DDS[46] | Thai | Thailand | hospital outpatient clinic | 30 |  |  | Elderly diabetes patients | 5 | gerontological nursing, geriatric medicine, diabetes, linguistics and culture, and instrument development |
| DDS[45] | English | Malaysia | primary care clinic | 5 |  |  |  | 3 | a family physician, a researcher familiar with validation of instruments, and a family medicine trainee |
| DDS[101] | Portuguese | Brazil | teaching hospital | 40 |  | both genders | T2DM, diagnosed >1 year ago |  |  |
| DDS[102] | Bahasa Indonesia | Indonesia | hospital | 10 | 65 (7) | 30% | retired civil servants, 7 oral therapy, 3 oral medication and insulin |  |  |
| DDS[103] | Polish | Poland |  | 10 |  |  | T2DM |  |  |
| SADDS[47] | Arabic | Saudi Arabia |  | 30 |  |  | diabetes |  |  |
| DFS-SF[52] | Chinese | China | hospital | 6 |  |  | DFU |  |  |
| DHP-18[104] | Spanish | Ecuador |  | 8 |  |  | T2DM |  |  |
| Diabetes Questionnaire [35] | Swedish | Sweden |  | 3 |  |  | T1DM (1), T2DM (2) | 6 | physicians (n = 2), nurses (n = 2), researchers (n = 2) |
| Diabetes-39[105] | Arabic | Jordan |  | 30 |  |  |  |  |  |
| Diabetes-39[106] | Vietnamese | Vietnam |  | 10 |  |  |  |  |  |
| Diabetes-39[107] | Portuguese | Brazil | basic health service | 4 |  |  |  |  |  |
| DIMS[108] | Chinese | China |  | 0 |  |  |  | ? | colleagues |
| DMQoL[109] | Persian | Iran |  | 37 | 51 | 57% | diabetes |  |  |
| DQOL[54] | Iranian | Iran |  | 30 | 47 (13) | 43% | T2DM |  |  |
| DQOL[55] | Japanese | Japan |  | 10 |  |  | diabetes | 3 | bilingual physicians |
| DQOL[49] | Malay | Malaysia |  | 7 to 15 |  |  | diabetes |  |  |
| DQOL[110] | Taiwanese | Taiwan |  | 7 | 60-80 | 43% | diabetes |  |  |
| DQOL[56] | Turkish | Turkey |  | 45 |  |  | university students with T1DM or T2DM |  |  |
| DQOL[111] | Afaan Oromoo | Ethiopia |  | 30 |  |  | T2DM |  |  |
| PAID[112] | Korean | Korea |  | 20 | 60 (12) | 40% | T2DM, disease duration 7 (5) year |  |  |
| PAID-5[112] | Korean | Korea |  | 20 | 60 (12) | 40% | T2DM, disease duration 7 (5) year |  |  |
| QOLSID[96] | Iraq | Arabic |  | 10 |  |  | diabetes | 7 | 2 physicians, 2 DM educators, 2 pharmacists, 1 public health specialist |

T1DM = type I diabetes mellitus; T2DM = type II diabetes mellitus

**Appendix 7. Quality of content validity studies**

| **PROM** | **Language** | **Content validity** | | | | |
| --- | --- | --- | --- | --- | --- | --- |
|  |  | **Asking patients** | | | **Asking experts** | |
|  |  | **Relevance** | **Compre-hensiveness** | **Compre-hensibility** | **Relevance** | **Compre-hensiveness** |
| DCP[100] | Chinese |  |  | D | D |  |
| DDRQOL-R[91] | Japanese |  |  | D | D | D |
| DDRQOL-R short form[91] | Japanese |  |  |  | D |  |
| DDS[46] | Thai |  |  | D | D |  |
| DDS[113] | English |  |  | D | ? | ? |
| DDS[101] | Portuguese |  |  | D |  |  |
| DDS[102] | Bahasa Indonesia |  |  | D |  |  |
| DDS[103] | Polish |  |  | D |  |  |
| SADDS-17[47] | Arabic | ? | ? | D |  |  |
| DFS-SF[52] | Chinese | I |  | D |  |  |
| DHP-18[104] | Spanish |  |  | D |  |  |
| Diabetes Questionnaire[35] | Swedish | D | D | D | D | D |
| Diabetes-39[105] | Arabic |  |  | D |  |  |
| Diabetes-39[106] | Vietnamese |  |  | D |  |  |
| Diabetes-39[107] | Portuguese |  |  | D |  |  |
| DIMS[108] | Chinese |  |  |  | ? | ? |
| DMQoL[109] | Persian |  |  | D |  |  |
| DQOL[54] | Iranian |  |  | D |  |  |
| DQOL[55] | Japanese | ? | ? | ? | ? | ? |
| DQOL[49] | Malay |  |  | D |  |  |
| DQOL[110] | Taiwanese |  |  | D |  |  |
| DQOL[56] | Turkish |  |  | D |  |  |
| DQOL[111] | Afaan Oromoo |  |  | D |  |  |
| PAID[112] | Korean |  |  | D |  |  |
| PAID-5[112] | Korean |  |  | D |  |  |
| QOLSID[96] | Arabic | D |  | D | D | D |

V = very good; A = adequate; D = doubtful; I = inadequate. Empty cells indicate that no study on these aspects are available.
